# Comparative study between two types of *Crocus sativus* L. corms: chemical composition, antioxidant, genotoxic, and cytotoxic effects

**DOI:** 10.1186/s40643-025-00889-2

**Published:** 2025-10-10

**Authors:** Sanae Baddaoui, Ennouamane Saalaoui, Samira Mamri, Sabir Ouahhoud, Diego Salagre, Anas Ziani, Amine Khoulati, Abdelkrim Abousalham, Bassem Jaouadi, Abdeslam Asehraou, Ramzi A. Mothana, Hanan M. Al-Yousef, Ahmad Agil

**Affiliations:** 1 Laboratoire de Bioressources, Biotechnologie, Ethnopharmacologie et Santé (LBBES), Faculté des Sciences d’Oujda (FSO), Université Mohammed Premier (UMP), Bd Mohamed VI, BP 717, 60000 Oujda, Morocco; 2 Department of Pharmacology, BioHealth Institute Granada (IBs Granada), Neuroscience Institute (CIBM), School of Medicine, University of Granada, 18016 Granada, Spain; 3 Laboratory of Biological Engineering, Faculty of Sciences and Technology, University Sultan Moulay Slimane, 23000 Beni Mellal, Morocco; 4 Faculty of Medicine and Pharmacy, University Sultan Moulay Slimane, 23000 Beni Mellal, Morocco; 5 Faculty of Medicine and Pharmacy University Mouhamed I , BP 724, 60000 Oujda, Morocco; 6 Univ Lyon, Université Lyon 1, Institut de Chimie et de Biochimie Moléculaires et Supramoléculaires (ICBMS), UMR 5246 CNRS, Génie Enzymatique, Membranes Biomimétiques et Assemblages Supramoléculaires (GEMBAS), Bât Raulin, 43 Bd du 11 Novembre 1918, 69622 Villeurbanne Cedex, France; 7 Laboratoire des Biotechnologies Microbiennes et Enzymatiques et de Biomolécules (LBMEB), Centre de Biotechnologie de Sfax (CBS), Université de Sfax (USF), Route de Sidi Mansour Km 6, BP 1177, 3018 Sfax, Tunisia; 8 Department of Pharmacognosy, College of Pharmacy, King Saud University, 11451 Riyadh, Saudi Arabia

**Keywords:** *Crocus sativus* L., Corms, Bioactive molecules, Antioxidant activity, Genotoxicity, Cytotoxicity

## Abstract

Saffron (Crocus sativus L.) corms, often discarded due to their small size, represent a valuable by-product with potential health benefits. This study aims to enhance the value of saffron corms by comparing stored corms (HEES) and fresh corms (HEEF) in terms of chemical composition, antioxidant, genotoxic, and cytotoxic effects. Both corm types were macerated in 50% ethanol, and levels of polyphenols, flavonoids, carotenoids, lycopene, anthocyanins, saponins, and sugars were quantified. Their antioxidant capacity was assessed through DPPH, FRAP, and β-carotene assays, while genotoxicity was evaluated via comet assays, and cytotoxicity was tested on CCD18 normal colon cells using MTT and crystal violet assays. Results showed that stored corms contained higher levels of phenolic compounds (0.781 ± 0.42 µg GAE/mg extract), flavonoids (1.13 ± 0.64 µg QE/mg extract), and carotenoids (27.99 µg β-carotene/g dry matter), compared to fresh corms. HEES also exhibited stronger antioxidant activity with an IC50 of 169.57 µg/mL in the DPPH assay, while HEEF showed an IC50 of 434.37 µg/mL. Both extracts displayed genotoxicity at 50 µg/mL and cytotoxicity on normal colon cells (CCD18) at approximately 300 µg/mL. Stored saffron corms are a rich source of bioactive molecules and exhibit greater antioxidant activity compared to fresh corms. However, both extracts demonstrate genotoxic and cytotoxic effects at higher doses, emphasizing the need for careful evaluation of their potential therapeutic applications. These findings suggest that saffron corms, particularly those that are stored, could be valuable in health-related fields but warrant further investigation.

**Graphical Abstract:**

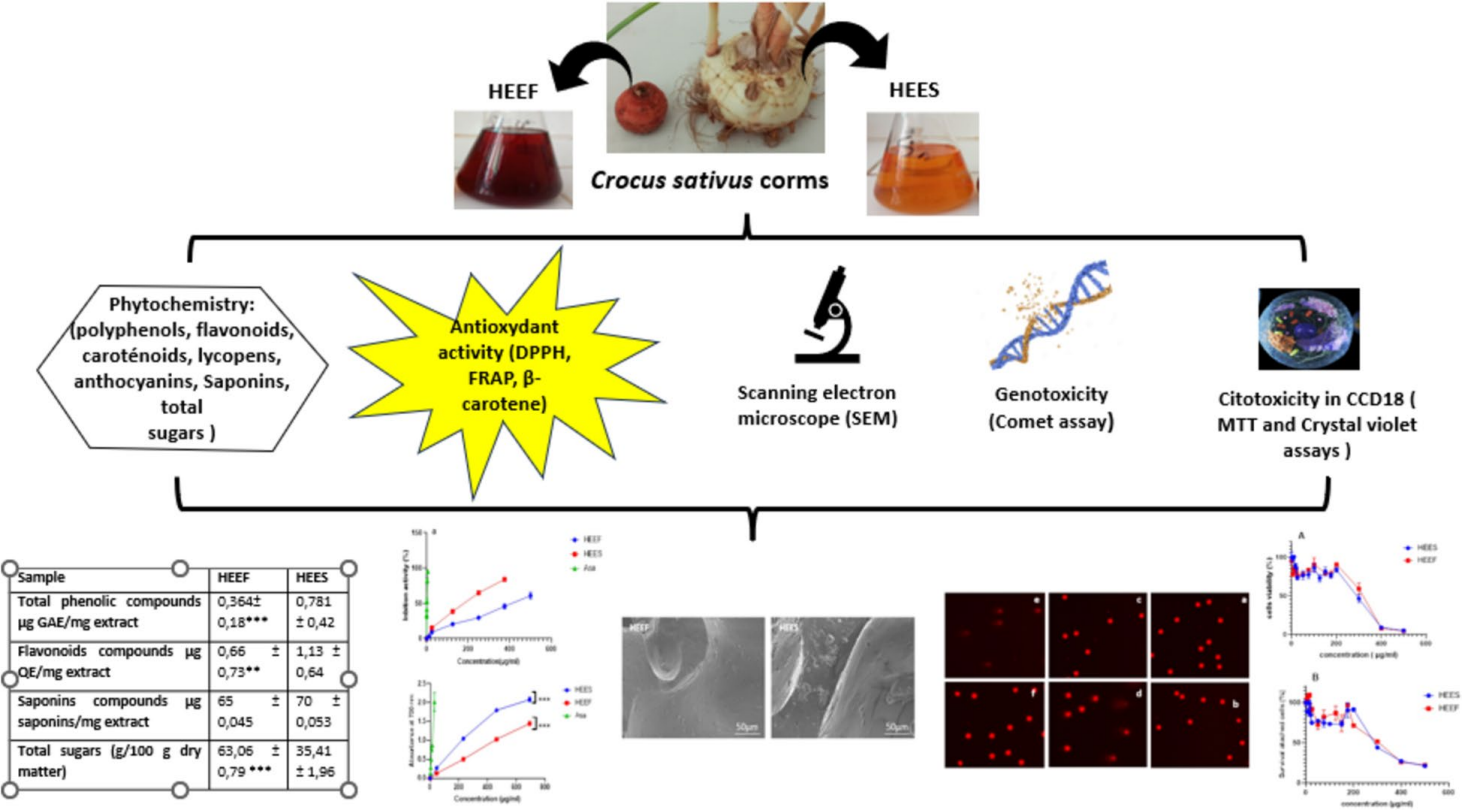

## Introduction

*Crocus sativus* L. is an aromatic and medicinal plant that belongs to the Iridaceae family, and it is well-known for the spice saffron, which is obtained from the stigmas of this plant (Wu et al. 2023). However, the production of 1 kg of this prized saffron generates significant by-products, including approximately 35 kg of tepals, 1500 kg of leaves, and numerous corms (100 kg), which, owing to their small size, are unsuitable for grinding or commercial sale (Sánchez-Vioque et al. 2012). Nevertheless, numerous researchers have concentrated on enhancing the value of saffron by-products (Esmaeelian et al. 2024a), in divers pharmacological effects including the scavenging of reactive oxygen species (ROS), and DNA protective effect (Ouahhoud et al. 2022), and protective effect against gentamicin-induced nephrotoxicity in rat kidney (Mamri et al. 2022), anti-inflammation (Yang et al. 2024), hepatoprotective (Omidi et al. 2014), antihypertensive (Butnariu et al. 2022), antiobesity (Bursać et al. 2024), antidiabetes (Ouahhoud et al. 2019), antidepression (Wang et al. 2010), and antimicrobial activities (Esmaeelian et al. 2021), these effects are attributed to some bioactive molecules that include polyphenols, flavonoids, saponins, and carotenoids (Wu et al. 2023b).

*Crocus sativus L*.’s corms are the underground stems of the plant that are rich in fatty, and amino acids, and carbohydrate substances. (Chrungoo and Farooq 1985; Bagri et al. 2017), phenolic compounds (Esmaeili et al. 2011; Sánchez-Vioque et al. 2016), and crocetin (Rubio-Moraga et al. 2010). Additionally, there are two known oleanane-type triterpenoid saponins: azafrine 1 and azafrine 2. (Rubio-Moraga et al. 2011).

Several studies have shown that Crocus sativus L. corms offer numerous pharmacological benefits, including antioxidant activity (Esmaeili et al. 2011; Baba et al. 2015; Lahmass et al. 2018; Esmaeelian et al. 2021), and antidepressant effects from their bioactive components (Wang et al. 2010). Similarly, they reveal good metal-chelating (Sánchez-Vioque et al. 2012), and antibacterial activities (Esmaeelian et al. 2021). Also, the antifungal effect of saponins detected on the outer part of the *Crocus sativus* L. corms, against *Fusarium oxysporum*, *Bipolaris spicifera*, and *Aspergillus* Niger, has been proven (Rubio-Moraga et al. 2013). The saponins found in saffron corms have been shown to effectively inhibit both the expression and secretion of genes related to pro-inflammatory cytokines, (Keller et al. 2021). Furthermore, the extract of triterpenoid saponins (GS5) from Crocus sativus L. corms has shown cytotoxic effects on HeLa tumor cells (Rubio-Moraga et al. 2011), also as well as a proteoglycan isolated from saffron corms has shown a cytotoxic effect against human cervical epithelioid carcinoma cells (Escribano et al. 1996, 2000).

Therefore, this study seeks to enhance the value of *Crocus sativus* L. corms by conducting a comparative analysis of two distinct types, labeled HEES and HEEF. Our objective was to identify potential applications for these small, stored corms.

## Materials and methods

### Plant material

Two types of *Crocus sativus* L. corms HEES and HEEF originated from Talioun (Morocco) were used in this study, corms collected in January, 2022 and stored in the dark at room temperature 25 ± 2 °C (one year), and other corms collected in January, 2023. The plant species has been registered in the botanical garden of Mohammed Premier University, Oujda, Morocco, under voucher number HUMPOM210.

### Extraction

The two types of corms were washed, dried in an oven (37 °C) for 24 h, and ground using an automatic grinder, then extracted by maceration of 10 g of corm powder in 100 mL of the mixture (50% ethanol in water) for 24 h (sorting times), the extract was filtered and the solvent was vaporized in an oven at 38 °C for 24 h, finally the dry extract was preserved at − 20 °C until use.

### Chemical composition *Crocus sativus* L. corms

#### Quantification of total polyphenol content

The Folin-Ciocalteu technique was used to measure total polyphenols (Tp). 200 µL of the Folin-Ciocalteu reagent and 1.35 mL of distilled water were mixed with 50 µL of each of the two hydroethanolic extracts (HEES, HEEF). After 3 min, 400 *µ*L of 20% sodium carbonate was added to the mixture, carefully vortexed and incubated for twenty minutes at 30 °C in a water bath. The absorbance was measured at 760 nm. The standard range used gallic acid (GA) in concentrations ranging from 25 to 1000 µg/mL in methanol. For each milligram of dry matter, the Tp content was reported as µg of gallic acid equivalent (Tian et al. 2011).

#### Total flavonoid content determination

Total flavonoids (FT) were determined using a colorimetric method based on 250 *µ*L of the extracts (HEES and HEEF) were amalgamated with 500 *µ*L of distilled water and adjusted with 750 µL of aluminum chloride (2%). After vortexing the mixture, the absorbance at 420 nm was determined. A standard range was performed using quercitrin at concentrations of 0.00625; 0.0125; 0.025, 0.05, and 1 mg/mL (dissolved in methanol). The expression for the total flavonoid content was µg quercetin per milligram of extract. Every test is run through a minimum of three times. Each test is repeated at least three times (Ayoola et al. 2008).

#### Determination of total carotenoids

2 g of corm powder was combined with 20 mL of (hexane/acetone/ethanol at 2*v*/*v* /*v*, respectively), the solution was centrifuged at 250 rpm for 20 min, the solvent was recovered, however the pellet was mixed with 10 mL of hexane, centrifuged for 20 min at 250 rpm, and finally the reading was taken at 420 nm. Carotenoids content was calculated using β-carotene as a standard (Sass-Kiss et al. 2005).

#### Determination of anthocyanins

The method previously mentioned was used to measure the anthocyanin content (Ganjewala et al. 2008). After mixing 10 mL of 0.1 N HCl (in methanol), with around 1 g of dried corms, stirring for 30 min, and centrifuging for 20 min at 5000 rpm, the mixture was removed. 0.5 mL of 0.1 N HCl in methanol was combined with a 0.1 mL aliquot of the supernatant. After measuring the absorbance at 530 nm, the milligram equivalents of cyanidin 3-glucoside (mg EqCG/100 g dry matter) were calculated using the following formula:

1 $$\text{Anthocyanine }\left(\text{mg}\frac{\text{EqCG}}{100\text{ gMS}}\right)=\frac{\text{Abs }530\text{ nn }\times \text{ MM }\times \text{ DF }\times 1000}{\upvarepsilon }$$

MM: cyanidin 3-glucoside’s molar weight (449.2 g/mol), ε: Cyanidin 3-glucoside's molar extinction coefficient, 38,000 L. mol^−1^.cm^−1^, DF: dilution factor

#### Determining lycopene content

The technique described by Rodriguez-Amaya (2001) was used to determine the lycopene content. The procedure involved mixing 0.1 g of corms with 10 mL of (hexane, acetone, and ethanol in a 50:50:50 v/v ratio), homogenizing the mixture for 10 min, then centrifuging it for 15 min at 5000 rpm. After dilution with 10 mL of hexane, approximately 1 mL of the recovered extract was tested for absorbance at 472 nm. The amount of lycopene is reported using the formula below:

2 $$\text{Lycopene}\left(\frac{\text{mg}}{100\text{ gDM}}\right)=\frac{\text{ Abs }472 \times \text{ F }\times {10}^{6} \times \text{ V}}{\left(3450 \times 100 \times \text{ W}\right)}$$

DF, V, and W: refers to the dilution factor, volume and weight, respectively.

#### Determination of saponin

For both fresh and stored Crocus sativus L. corms, the amount of saponin was measured in dried hydroethanolic extracts. To 100 µL of the extracts (HEES and HEEF), 100 µL aliquot of 0.8% vanillin in ethanol and 1000 µL of 72% sulfuric acid were added. Before cooling on ice for five minutes, the blend was vortexed and heated to 60 °C for 10 min. The absorbance was measured at 520 nm, as described by Le et al. (2018) and the total saponin concentration was determined using a standard saponin calibration curve.

#### Determination of sugars

Total sugar content was determined using the method of (Bachelier and Gavinelli 1966) employing anthrone and sulfuric acid as reagents. A solution of 1 g of anthrone dissolved in 500 mL of 95% sulfuric acid was prepared. To 2.5 mL of *Crocus sativus* L. corm extract, 5 mL of the anthrone-sulfuric acid solution was added. After mixing, the tubes were incubated at 50 °C for 10 min, and absorbance was determined at 620 nm. The total sugar content was expressed as grams of glucose per gram of dry matter.

#### Scanning electron microscopy

Scanning electron microscopy (SEM) images of fresh and stored Crocus sativus corms were obtained using a Thermo Scientific™ Quattro ESEM system (Thermo Fisher Scientific, Paisley, UK) at an acceleration voltage of 15 kV. To enhance conductivity for the electron beam, the samples were coated with a thin layer of gold. Energy-dispersive X-ray (EDX) spectra were collected with a detector integrated into the same SEM instrument (Aaddouz et al. 2023).

### Antioxidant activity

#### Ferric reducing antioxidant power (FRAP)

To get the reducing power, 1 mL of each of the extracts (HEES and HEEF) was mixed with 2.5 mL of phosphate buffer (0.2 M, pH 6.6) and 2.5 mL of a 1% potassium ferricyanide (K₃Fe(CN)₆). At 50 °C, the reaction solution was incubated for 20 min. To halt the process, 2.5 mL of 10% trichloroacetic acid (TCA) was added after the incubation period. Following that, the amalgam was centrifuged at 3000 rpm for 30 min. A fresh 0.1% FeCl₃ solution (500 µL) was then combined with 2.5 mL of distilled water and 2.5 mL of the supernatant. Ascorbic acid was used as the reference standard due to its well-documented reducing capacity and its widespread use in the FRAP assay as a benchmark antioxidant. 700 nm was used to measure absorbance (Karagözler et al. 2008).

#### 2,2-diphényl-1-picrylhydrazyl (DPPH)

With some slight adjustments, the approach of Sánchez‐Moreno et al. (1998) was used to assess the DPPH-radical scavenging activity. The procedure involved mixing 50 μL of Crocus sativus L. corms extract HEES and HEEF or standard with 1950 μL of DPPH solution (2.4 mg of DPPH diluted in 100 mL methanol). The absorbance was measured at 517 nm following a half-hour dark incubation period. Ascorbic acid was used as the reference standard due to its high radical scavenging ability and frequent use as a positive control in DPPH assays to evaluate antioxidant potential. The following formula was used to determine the percentage of inhibition:

3 $$\text{Percentage of inhibition}= \left[\frac{A0 - Af}{A0}\right]\times 100$$

A₀: DPPH absorbance in the absence of extracts.

Af: DPPH absorbance in the presence of extracts or standard.

#### β-carotene bleach test

The assay for β-carotene bleaching was carried out using the methodology described by Kabouche et al. (2007). In summary, 200 mg of Tween 80 and 20 mg of linoleic acid were combined with β-carotene (2 mg) that had been suspended in 1 mL of chloroform, and the chloroform was then evaporated at 35 °C using a rotating evaporator. Next, 2.45 mL of the β-carotene/linoleic acid emulsion was added to tubes that contained 50 µL of the extracts. Absorbance was measured at 490 nm both initially (A0) and after (Af) incubation at 50 °C for two hours, using a blank sample that contained distilled water and the extract at the same concentration. BHT (butylated hydroxytoluene) was used as the reference standard due to its well-known lipid peroxidation inhibitory properties and its widespread use as a positive control in β-carotene bleaching assays. The proportion of β-carotene oxidation was computed using the following formula:

4 $${\text{Percentage of oxidized}} \beta - {\text{carotene}} = \left[ {\frac{A0 - Af}{{A0}}} \right] \times 100$$

### Genotoxic effect

#### Comet assay

With minor adjustments, the comet assay used in this investigation was based on the Ouahhoud et al. 2022 technique. After pentobarbital anesthesia, blood samples were collected from the retroorbital vein of Wistar rats. Then, 400 μL of the PBS-diluted extract (HEES or HEEF) at concentrations of 5, 10, 25, and 50 μg/mL were added. For the positive control, 400 μL of a 250 mM solution of H_2_O_2_ was used. This concentration was selected after testing a range of H₂O₂ doses to determine the genotoxic concentration under our experimental conditions, while the control group (CG) received only 400 μL of PBS. The samples were then incubated with 10 μL of cells from the blood for one hour at 37 °C. After that, the resulting mixture was centrifuged for 10 min at 4500 rpm. The pellet was again suspended in 1 mL of PBS after the supernatant was disposed of. There were three iterations of this washing process. After the final centrifugation, 200 μL of 0.5% low melting point (LMP) agarose (prepared in PBS at 37 °C) was added to the pellet. The mixture was spread onto a slide containing a first layer of agarose and quickly covered with a coverslip to form a second agarose layer. Next, after cooling for 5 min, the slides were submerged in lysis buffer after the coverslips had been eliminated. (20 mM Tris, 100 mM Na2-EDTA, 1% *N*-lauroylsarcosine sodium, 300 mM NaOH, 2.5 M NaCl, 10% DMSO, and 1% Triton X-100) for 1 h at 4 °C in the dark. After that, double-distilled water was used three times to rinse the slides. For DNA unwinding, the slides were incubated in electrophoresis solution 300 mM NaOH and 1 mM Na2-EDTA, (pH 13; 20 min). Electrophoresis was conducted for 20 min at a constant voltage of 20 V and 300 mA at 4 °C. After electrophoresis, the slides were submerged for 5 min in neutralization buffer (400 mM Tris, pH 7.5). To eliminate the electrophoresis solution and restore DNA strand pairing, this procedure was carried out three times. After three distilled water rinses, ethidium bromide was employed to stain the slides. They were then examined and imaged using the ZOE Cell Imager. DNA damage was quantified using an image analysis tool integrated with Comet Assay IV software.

### Cytotoxic effect on CCD18 colon normal cells

#### Preparation of the cells culture

One liter of Dulbecco’s Minimum Essential Medium (DMEM) culture medium, supplemented with 10% fetal calf serum (FBS), 1% penicillin/streptomycin, and 1% l-glutamine, is used to cultivate the cell lines (normal colon cells, CCD18). The nutrients needed for human cell growth and maintenance are abundant in DMEM. The cells were kept at 37 °C in a humidified environment with 5% CO_2_ to guarantee their survival.

#### MTT assay

After 24 h, the cytotoxic effect of corm extracts on the viability of CCD18 normal colon cells was assessed using the MTT test (3-(4,5-dimethylthiazol-2-yl)-2,5-diphenyltetrazolium bromide). By measuring how mitochondrial dehydrogenases in metabolically active cells reduce MTT, a yellow tetrazolium salt, into formazan crystals, this assay assesses cell viability. The concentrations of the extracts that were examined ranged from 5 to 500 µg/mL. Following the exposure period, 200 μL of fresh media was added to the culture medium, along with 20 μL of MTT reagent (5 mg/mL in PBS). For four hours, the cells were cultivated at 37 °C. To dissolve the formazan crystals, 100 μL of DMSO was added after the medium was removed. The percentage of live cells was determined by measuring absorbance at 540 nm using a microplate reader (Tolosa et al. 2015).

#### Crystal violet assay

After treating the cells with corm extracts at concentrations of 5 to 500 µg/mL for 24 h, cell viability was assessed using the crystal violet assay. After taking off the culture medium, each well received 40 µL of crystal violet solution (0.75 g crystal violet in 120 mL distilled water and 30 mL methanol). Following a 20-min shake, the crystal violet solution was removed, and distilled water was used three times to properly clean the microplate. Each well was then filled with 160 µL of methanol to dissolve the dye. By measuring absorbance at 570 nm, the percentage of live adherent cells was determined (Feoktistova et al. 2016).

### Statistical analysis

The data was statistically evaluated using a one-way ANOVA with significance levels set at P < 0.05, P < 0.01, and P < 0.001 using GraphPad Prism 8.0 software. All analyses were performed three times.

## Results

### Chemical composition *Crocus sativus* L. corms

Tables 1 and 2 show that stocked corms (HEES) have the highest contents of phenolic (0.781 ± 0.42) µg GAE/mg extract, flavonoids (1.13 ± 0.64) µg QE/mg extract, carotenoids (27.99 ± 0.034) µg β-carotene /g dry matter, lycopene (0.105 ± 0.0081) mg/ 100 g dry matter, and anthocyanins (5.24 ± 0.34) µg /mg dry matter, compared to fresh corms (HEEF). Additionally, total sugar content was higher in HEEF (63.06 ± 0.79) g/100 g dry matter, than in HEES (35.41 ± 1.96) g/100 g dry matter. However, when comparing the saponin content of HEEF (65 ± 0.045) µg saponins/mg extract and HEES (70 ± 0.053) µg saponins/mg extract, there was no significant distinction (P > 0.05).

**Table 1:** Total phenolic, flavonoids, anthocyanin, saponin, and total sugar content in *Crocus sativus* L. corms extracts HEES and HEEF

Sample	HEEF	HEES
Total phenolic compounds µg GAE/mg extract	0.364 ± 0.18***	0.781 ± 0.42
Flavonoids compounds µg QE/mg extract	0.66 ± 0.73**	1.13 ± 0.64
Saponins compounds µg saponins/mg extract	65 ± 0.045	70 ± 0.053
Total sugars (g/100 g dry matter)	63.06 ± 0.79 ***	35.41 ± 1.96

HEES: hydroethanolic extract of stocked corms, HEEF: hydroethanolic extract of fresh corms, GAE: galic acid equivalent, QE: quercetine equivalent, * (P < 0.05), **(P < 0.001), ***(P < 0.001), Significantly different from stokced corms

**Table 2:** Carotenoid, Lycopene, and anthocyanin contents in *Crocus sativus* L. stocked, and fresh corms

Sample	Carotenoid content µg β-carotene /g dry matter	Lycopene content (mg/ 100 g dry matter)	Anthocyanins content (µg /mg dry matter)
Stokced corms (dry matter)	27.99 ± 0.034	0.105 ± 0.0081	5.24 ± 0.34
Fresh corms (dry matter)	5.66 ± 0.009**	0.034 ± 0.0045**	1.24 ± 0.034**

* (P < 0.05), ** (P < 0.001), Significantly different from stocked corms

### Scanning electron microscope (SEM)

**Fig. 1:**
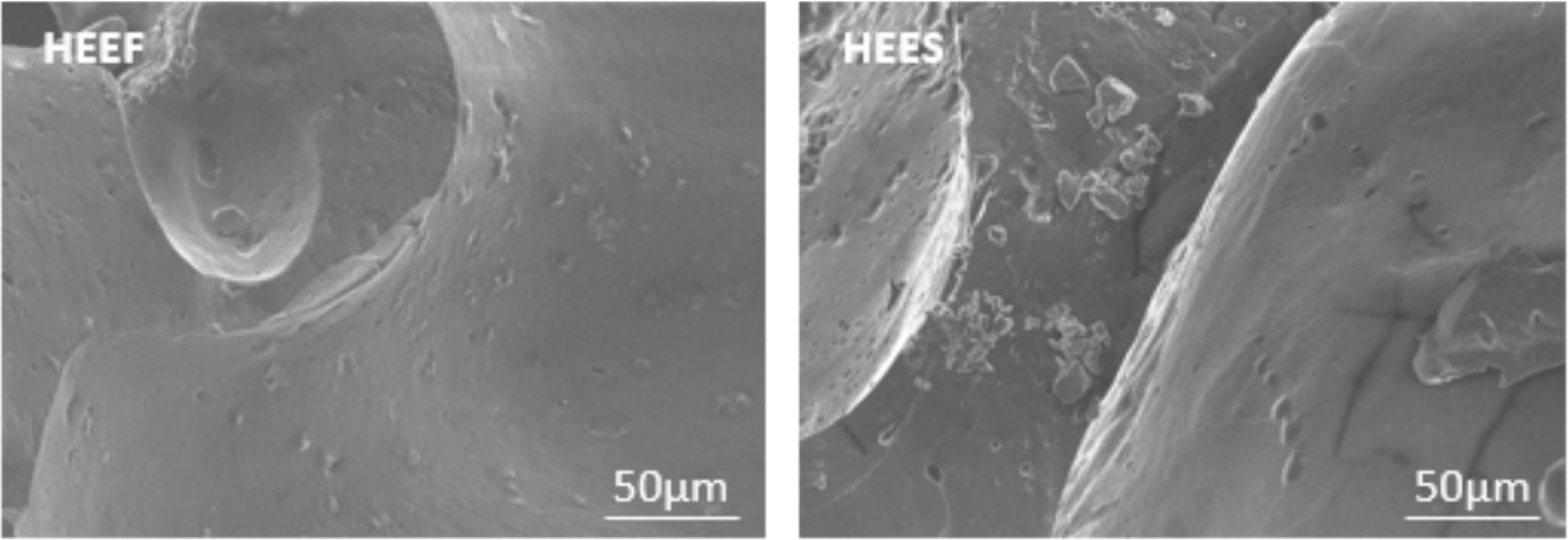
SEM image of *Crocus sativus* L. corms extract. HEEF: hydroethanolic extract of fresh corms. HEES: hydroethanolic extract of stocked corms

**Fig. 2:**
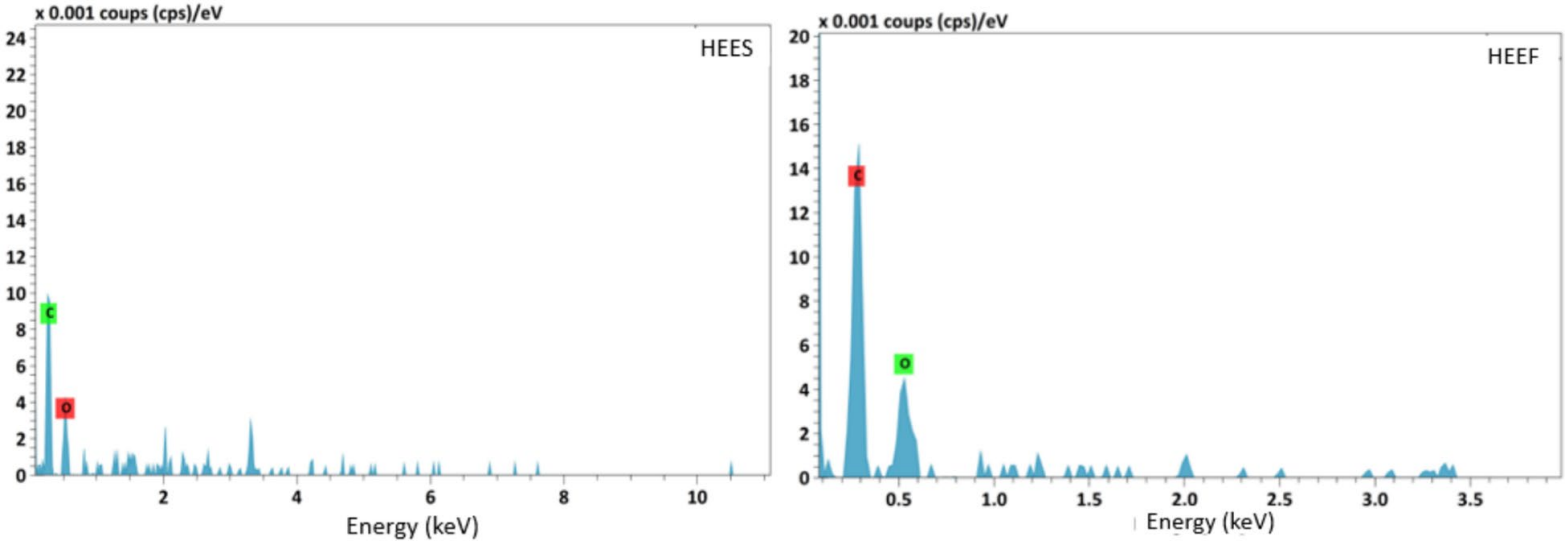
Energy Dispersive X-ray diffractive (EDX) Analysis showing the chemical element of *Crocus sativus* L. corms extract. HEEF: hydroethanolic extract of fresh corms, HEES: hydroethanolic extract of stocked corms

The analysis of Figs. 1 and 2, as well as Table 3, highlights differences between the two extracts, HEEF and HEES. Crystals are observed in the HEES extract compared to HEEF (Fig. 1), suggesting structural changes at the molecular level, possibly due to differences in storage. In addition, the carbon percentage is reduced and oxygen is augmented in HEES (Table 3), indicating chemical transformations, potentially involving the formation of new molecules containing other elements. The EDX spectra (Fig. 2) reveal that HEEF shows peaks between 0 and 3.5 keV, with maximum peaks corresponding to oxygen and carbon, suggesting that HEEF is primarily composed of organic molecules. In contrast, HEES presents peaks extending up to 10 keV, with the emergence of new peaks corresponding to other atoms. These findings confirm that storage significantly impacts the chemical composition of the extracts, leading to the formation of new molecules and the alteration of elemental proportions such as carbon and oxygen.

**Table 3:** Elemental composition results of *Crocus sativus* L. corms extract

Extract	Element	Normalized mass percent (%)
*HEES*	Carbon	64.53
	Oxygen	35.47
*HEEF*	Carbon	74.46
	Oxygen	25.54

HEEF: hydroethanolic extract of fresh corms, HEES: hydroethanolic extract of stocked corms

### Antioxidant activity

Examining the antioxidative potential of hydroethanolic extracts made from both freshly harvested and stored corms is the aim of this study. To achieve this objective, four tests are utilized: the DPPH analysis, the β-carotene analysis, the FRAP analysis, and the assessment of overall antioxidant activity. The findings provided in Table 4 demonstrate the anti-free radical activity of hydroethanolic extracts from fresh and stored corms. Ascorbic acid exhibited the highest activity (IC_50_ = 0.827 µg/mL), (P < 0.001) succeeded by HEES extract from stored corms (IC_50_ (HEES) = 169.57 µg/mL), and the HEEF extract from fresh corms demonstrated the least activity, with an IC_50_ value of 434.37 µg/mL. Concurrently, β-carotene oxidation inhibitory effect was evaluated in comparison to that of BHT (P < 0.001), which demonstrated the utmost effectiveness (IC_50_ = 16.54 µg/mL), surpassing both the extract from stored corms (IC_50_ = 43.54 µg/mL) and the extract from fresh corms (IC_50_ = 45.86 µg/mL).

**Table 4:** Ethanolic extracts of fresh and stock corms with IC50 values for β-carotene bleaching and DPPH radical scavenging

Sample	IC_50_ of DPPH (µg/mL)	IC_50_ of β-carotene (µg/mL)
Fresh corms (HEEF)	434.37 ± 0.23**^(###)^	45.80 ± 0.016 ^(###)^
Stocked corms (HEES)	169.57 ± 0.5 ^(###)^	43.45 ± 0.35 ^(###)^
Ascorbic acid	0.827 ± 0.82	ND
Butylated hydroxytoluene (BHT)	_ND	16.54 ± 0.0.09

As the positive control, ascorbic acid and BHT were used. The mean ± standard error of three measurements is used to present the results

** (P < 0.001), Significantly different from stocked corms, and ^###^(P < 0.001) Significantly different from ascorbic acid or BHT. ND. Not determined

**Fig. 3:**
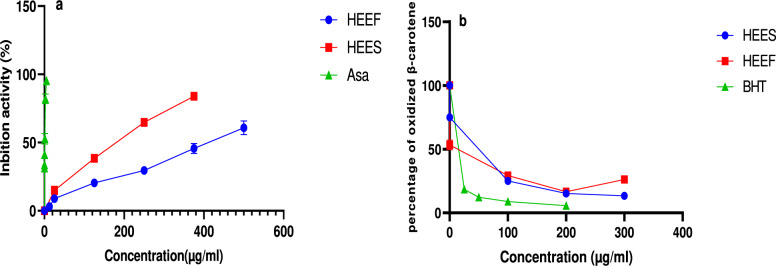
DPPH radical scavenging activities (**a**), and protective effect on β-carotene degradation (**b**), of two extracts HEES (Stocked corms), and HEEF (corms fresh). Values are expressed as means ± SEM (n = 3), Asa = ascorbic acid, BHT = butylhydroxytoluene. **(P < 0.001), ***(P < 0.001), Significantly different from ascorbic acid

Following the results illustrated in Fig. 3, extracts sourced from recently gathered and stored corms manifest not only a capacity to scavenge free radicals but also an ability to prevent β-carotene from oxidizing and an effect on the Ferric Reducing Antioxidant Power, these effects are dose dependent.

### Ferric reducing antioxidant power

**Fig. 4:**
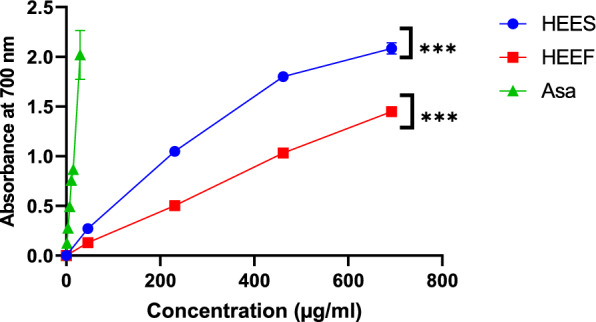
The ferric-reducing antioxidant power of two extracts: HEEF (fresh corms) and HEES (stocked corms). Means ± SEM are used to display the values (n = 3). Asa = Ascorbic acid. *** (P < 0.001), Significantly different from ascorbic acid

Ferric Reducing Antioxidant Power is focused on reducing of Fe3 + to Fe2 + by the antioxidant's electron transfer process. The results obtained show that the highest activity is contributed to ascorbic acid (P < 0.001) with an absorbance of 0.5 nm at a concentration of (7.15 ± 0.35) *µ*g/mL, Although the activity is higher in the HEES compared to the HEEF, the difference is not statistically significant (Fig. 4).

### Genotoxic effect on rat leukocytes

**Fig. 5:**
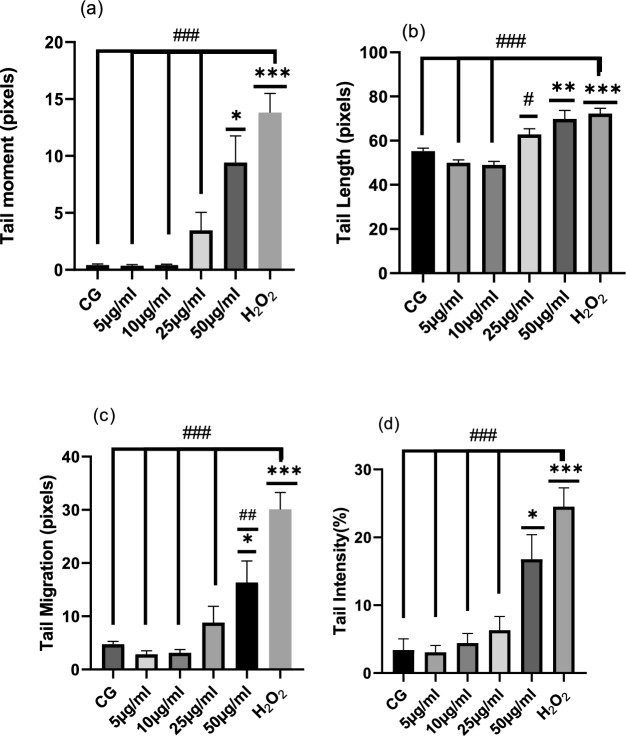
HEES’s genotoxic impact on rat leukocytes' tail moment (**a**), tail length (**b**), tail migration (**c**), and tail intensity (**d**). Mean ± SEM (50 cells × 2) is used to display the values. CG stands for control group, H_2_O_2_ for hydrogen peroxide, and HEEF for hydroethanolic extract from preserved corm. Significantly different from the control group (CG) are indicated by * (P < 0.05), ** (P < 0.001), and *** (P < 0.001), and significantly different from the H_2_O_2_ group are indicated by ^#^ (P < 0.05), ^##^ (P < 0.001), and ^###^ (P < 0.001)

**Fig. 6:**
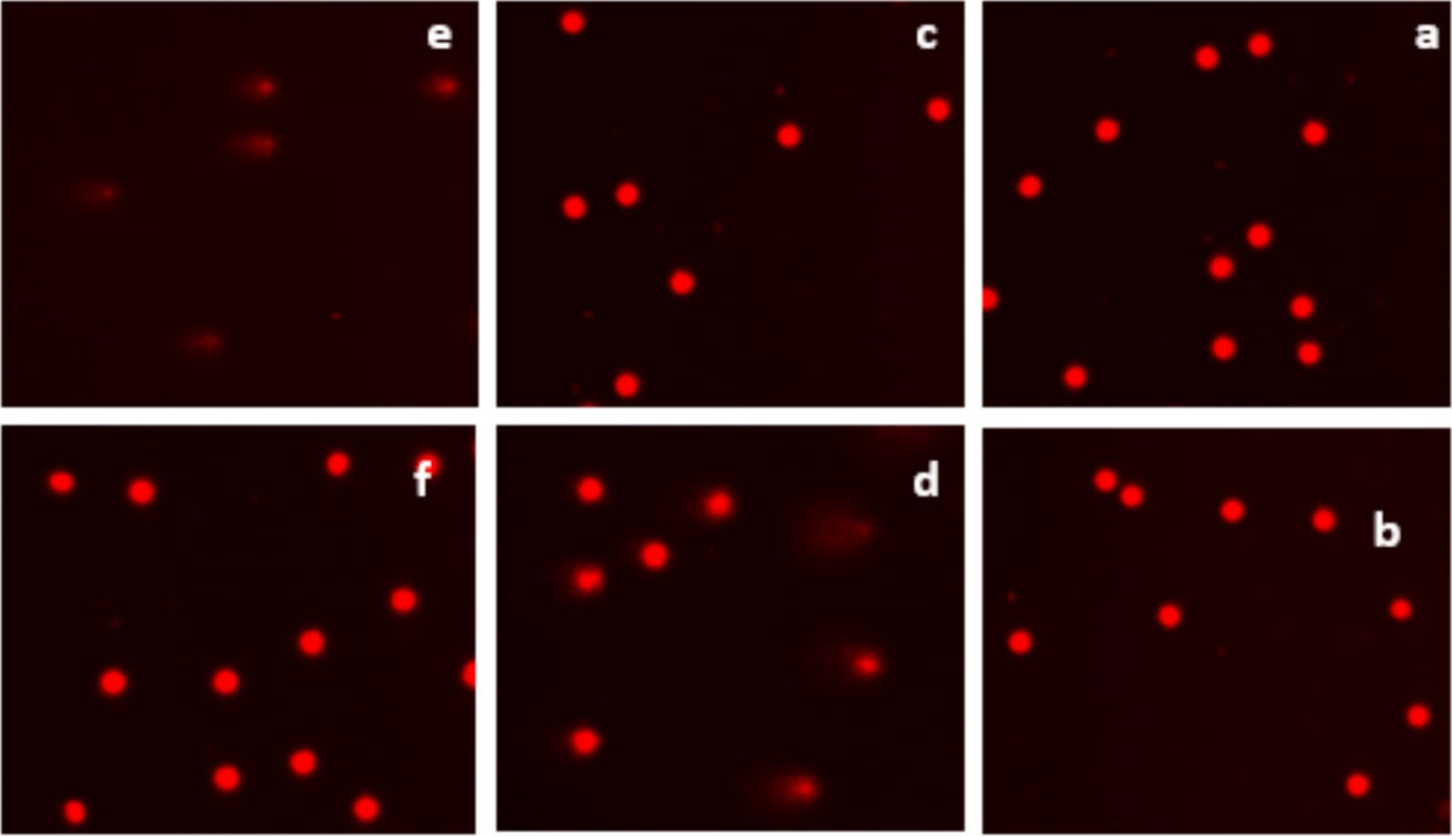
Various levels of DNA fragmentation detected by the comet assay following treatment with HEES extract from corms. **a** Cells treated with 5 µg/mL HEES, **b** cells treated with 10 µg/mL HEES, **c** cells treated with 25 µg/mL HEES, **d** cells treated with 50 µg/mL HEES, **e** cells treated with 250 mM H_2_O_2_, and **f** negative control, 400 × microscope objective

Figure 5 and 6 shows the effect of stored *Crocus sativus* corms on rat leukocyte DNA, the parameters analyzed are: tail moment, tail length, tail migration and tail intensity, demonstrating that HEES is nongenotoxic at 5, 10, and 25 µg/mL but is genotoxic at 50 µg/mL compared to the control groups (GC) with difference statistic significative, and no difference between the parameters of DNA cells treated with 50 µg/mL and H_2_O_2_ control positive group.

**Fig. 7:**
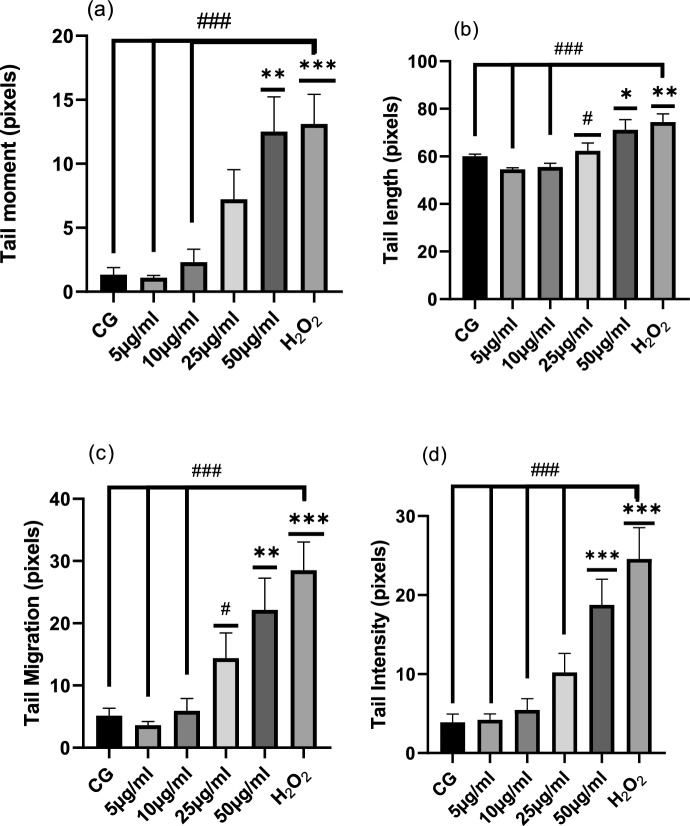
HEEF’s genotoxic effects on rat leukocytes' tail moment (**a**), tail length (**b**), tail migration (**c**), and tail intensity (**d**). The units of measurement are mean ± SEM (50 cells × 2). H_2_O_2_ stands for hydrogen peroxide, CG for control group, and HEEF for fresh corm hydroethanolic extract. * (P < 0.05), ** (P < 0.001), *** (P < 0.001), and ^#^ (P < 0.05), ^##^ (P < 0.001), and ^###^ (P < 0.001) indicate significant differences from the H_2_O_2_ group and the control group (CG), respectively

**Fig. 8:**
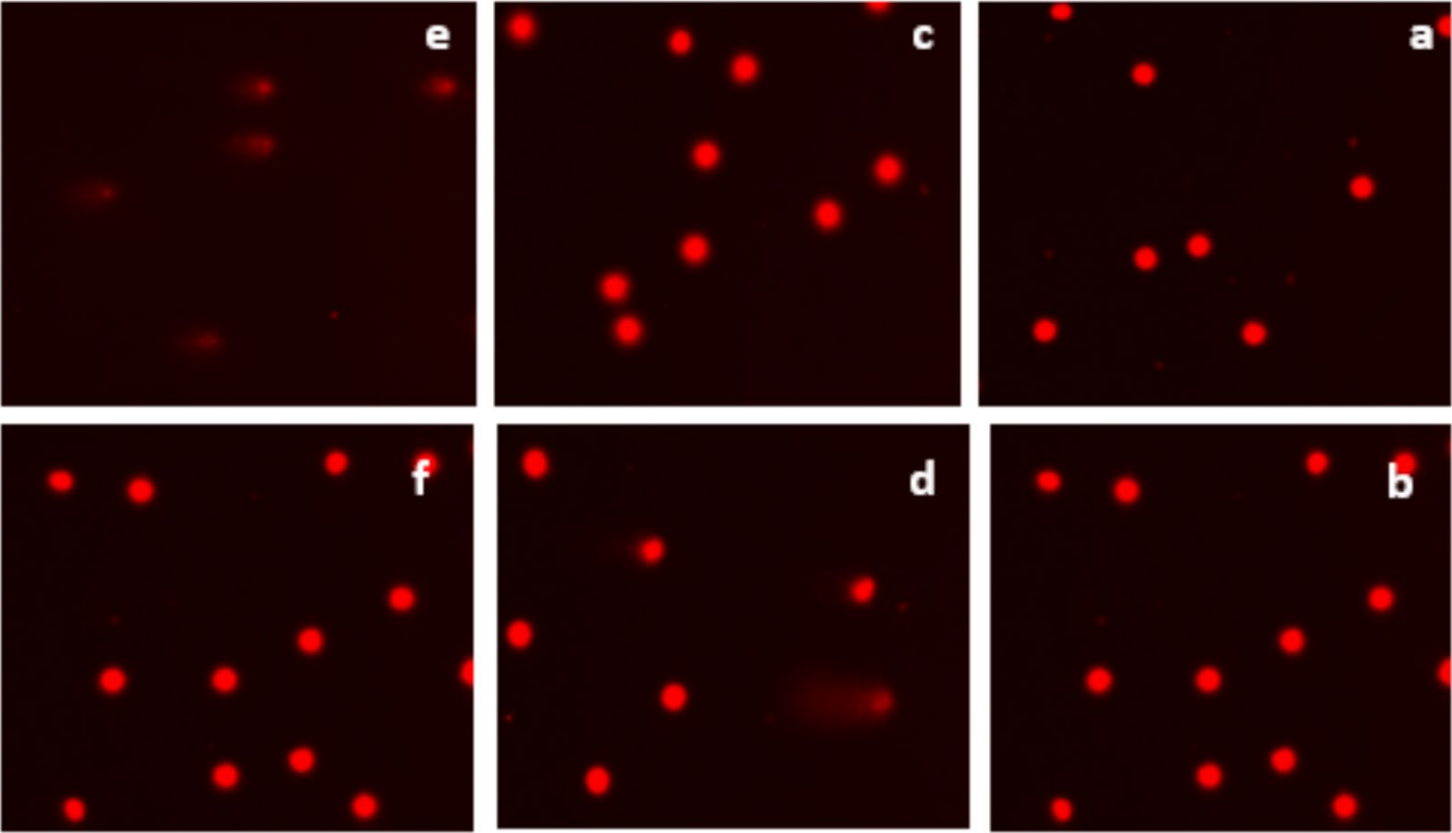
Various levels of DNA fragmentation detected by the comet assay following treatment with HEEF extract from corms. **a** Cells treated with 5 µg/mL HEEF, **b** cells treated with 10 µg/mL HEEF, **c** cells treated with 25 µg/mL HEEF, **d** cells treated with 50 µg/mL HEEF, **e** cells treated with 250 mM H_2_O_2_, and **f** negative control, 400 × microscope objective

Figures 7 and 8 illustrates the results of the effect of fresh *Crocus sativus* L. corms on rat leukocytes DNA, the parameters: tail moment, tail length, tail migration, and tail intensity which show this effect demonstrates that HEEF is genotoxic at 50 µg/mL compared to the control groups (CG), and no difference between the group treated with 50 µg/mL and H_2_O_2_ control positive group.

Figures 5 and 7 illustrate the same findings, showing that both fresh and stored corms exhibit genotoxicity at a concentration of 50 µg/mL. However, statistical analysis of comet assay parameters indicates that doses below 50 µg/mL do not produce a genotoxic effect when compared to the control group.

### Cytotoxicity of *Crocus sativus* corms in colon normal cells CCD18

**Fig. 9:**
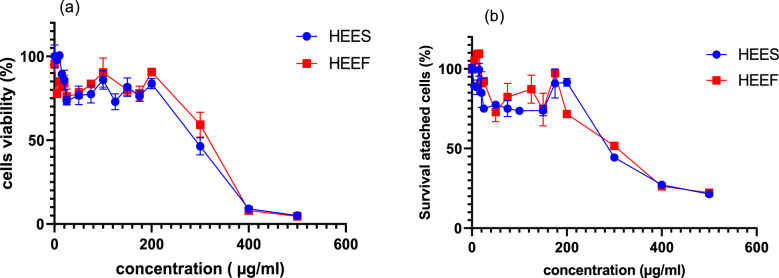
cytotoxic effect of HEEF and HEES on CCD18 colon normal cells, using MTT assay (**a**) and crystal violet assay (**b**). HEEF: hydroethanolic extract from fresh corms. HEES: hydroethanolic extract from stored corms

The results shown in Fig. 9 illustrate the cytotoxic effects of both stored (HEES) and fresh corms (HEEF), using two tests: the MTT and crystal violet tests. Both tests demonstrate that the two corms extract exhibit dose-dependent cytotoxicity on normal CCD18 colon cells, with a CC50 (HEES) = 312,5 ± 3,04 µg/mL, 339.0 ± 13.04 µg/mL and CC50 (HEEF) 315.1 ± 21.14 µg/mL, and 349.3 ± 5.28 µg/mL, determined by two test MTT and crystal violet respectively.

## Discussion

Our findings show that stored corms (HEES) had higher concentrations of phenolics (0.781 ± 0.42), flavonoids (1.13 ± 0.64), carotenoids (27.99 ± 0.034), lycopene (0.105 ± 0.0081), and anthocyanins (5.24 ± 0.34) compared to fresh corms (HEEF), which contained 0.364 ± 0.18, 0.66 ± 0.73, 5.66 ± 0.009, 0.034 ± 0.0045, and 1.24 ± 0.034, respectively. Additionally, total sugar content was higher in HEEF (63.06 ± 0.79) than in HEES (35.41 ± 1.96). However, when comparing the saponin content of HEEF (65 ± 0.045) and HEES (70 ± 0.053) µg/mg extract, there was no significant distinction. Several studies have demonstrated that Crocus sativus L. corms are a rich source of bioactive molecules, including polyphenols such as gallic acid, caffeic acid, and salicylic acid; carotenoids like crocetin; and saponins such as oleanolic acid and azafrin 1 and 2 (Esmaeili et al. 2011; Rubio-Moraga et al. 2011, 2013; Esmaeelian et al. 2024). The observed increase in bioactive molecules in stored corms may result from their synthesis in response to oxidative or environmental stress, such as dehydration or temperature fluctuations during storage. Verma and Shukla (2015) illustrated that the storage of plant secondary metabolites produced through biosynthetic pathways is influenced by biochemical, cellular, and developmental factors. Their concentrations are also affected by abiotic factors, including temperature, drought, salinity, light, UV radiation, and nutrient deficiencies. Additionally, sugars have been identified as key components of corm composition (Chrungoo and Farooq 1985; Bagri et al. 2017). In our study, we observed a decrease in sugar content in stored corms, likely due to their utilization as an energy source to sustain metabolic processes during storage. Similar findings have been reported in onions, where fresh bulbs contain higher carbohydrate levels than stored ones (*Allium cepa L*.) (Saviano et al. 2019). Wang et al. (2020) demonstrated that after 12 months of storage, enzymatic starch degradation led to a reduction in glucose, cellobiose, and lactulose levels, while saccharide derivatives increased. These changes influenced sugar transport and storage in reservoir cells, ultimately impacting the quality of brown rice.

DPPH, FRAP, and β-carotene were the three tests used to assess the antioxidant activity of HEES and HEEF corm extracts. These tests showed that corm extracts in the storage state had more potent antioxidant activity than fresh corm extracts, as illustrated in Figs. 1 and 2. With IC50 values of 169.57 ± 0.5 µg/mL as opposed to 434.37 ± 0.23 µg/mL for the HEEF extract, the HEES extract showed a higher ability to decrease the DPPH radical in the DPPH test. All of the extracts had lesser antioxidant activity than ascorbic acid, which served as a positive control and had an IC50 of 0.827 ± 0.82 µg/mL. Esmaeili et al. 2011 demonstrated that corms exhibit antioxidant activity, with IC_50_ values of 2055 ppm for waking corms and 8274 ppm for dormant corms, however Baba et al. 2015 the IC50 values for ethanolic extract of the corm, 246.22 ± 5.60, and water extract were found to be 465 ± 3.52. Similarly, the data of the FRAP assay in Fig. 4, revealed a higher iron-reducing capacity in the HEES extract compared to the HEEF extract, in comparison with (Baba et al. 2015) Corm ethanolic extracts exhibited greater action than their aqueous counterparts, however, (Sánchez-Vioque et al. 2012) demonstrate that the reducing power of the corm extract was nearly insignificant when compared to BHT control. the β-carotene test indicated an increase in the protective power of HEES against β-carotene oxidation compared to HEEF, though not significantly, with IC50 values of 43.45 ± 0.35 µg/mL for HEES and 45.80 ± 0.016 µg/mL for HEEF. Sánchez-Vioque et al. 2012, illustrate that at a concentration of 33 µg/mL the % of oxidized β-carotene is 40.0 ± 10.3. These results illustrate that the corms extract exhibits an antioxidant activity with a high effect is correlated with the high level of bioactive molecules (Wu et al. 2023b), where we found a significant quantity in stocked compared to fresh corms.

Therefore, to ensure the safe use of corm extracts, it is essential to identify their genotoxic and cytotoxic thresholds. To achieve this, we performed a comet assay to analyze genotoxicity, as well as MTT and crystal violet assays to evaluate cytotoxicity in normal colon cells CCD18.

Single gel electrophoresis, or the Comet assay, is a method utilized to assess DNA damage, that includes single and double-strand breaks, as well as oxidative damage induced by genotoxic agents (Cordelli et al. 2021) with four parameters tail length, tail moment, tail intensity, and tail migration. In the current research, the effects of treating blood cells with HEES and HEEF at doses of 5, 10, and 25 µg/mL were evaluated. Results indicated no significant effects on tail intensity, length, moment, or migration compared to the negative group. However, both extracts exhibited genotoxicity at a concentration of 50 µg/mL, so we can conclude that corms extract HEES, and HEEF were genotoxic over a dose of 50 µg/mL. Although, the cytotoxic effect in CCD 18 normal colon cells of both extract HEES, and HHEF demonstrate that the CC50 of HHES and HEEF determined by two test MTT and crystal violet: CC50 (HEES) = 312.5 ± 3.04 µg/mL, 339.0 ± 13.04 µg/mL and CC50 (HEEF) 315.1 ± 21.14 µg/mL, and 349.3 ± 5.28 µg/mL, respectively. We observe that there is no significant statistical difference between the HEES and HEEF extracts regarding their genotoxic dose (50 µg/mL) and they displayed CC_50_ cytotoxicity in normal colon cells CCD18 around 300 µg/mL. Despite the differences in their concentrations of bioactive molecules such as polyphenols, flavonoids, carotenoids, and anthocyanins, which are known for their antioxidant activity, both extracts exhibit similar properties. (Esmaeili et al. 2011) and are prooxidant and to have toxic effects at high doses (Madariaga-Mazón et al. 2019). Given that both extracts exhibit genotoxicity and cytotoxicity at the same doses, we hypothesize that this effect might be due to other compounds, specifically saponins, (Rubio-Moraga et al. 2011) that the two extracts have the same concentration of saponins.

Wide studies demonstrate that saponins exert many medicinal properties, such as anti-inflammatory, vasoprotective, hypocholesterolemia, immunomodulatory, hypoglycaemic, molluscicidal, antifungal, antiparasitic, and anticancer activity (Podolak et al. 2010; Rubio-Moraga et al. 2013; Kalachaveedu et al. 2015; Sánchez-Vioque et al. 2016; Almuzaini et al. 2024) but saponins are toxic at high doses (Esmaeelian et al. 2024a). Additionally, when saponins are injected intravenously, they can cause hemolysis of erythrocytes, by interacting with sterols in the erythrocyte membrane, this interaction increases membrane permeability leading to loss of hemoglobin (Marrelli et al. 2016). Brahmi saponin has a genotoxic effect on human lymphocytes at a dose of 30 µg/mL (Kalachaveedu et al. 2015).

At present, it is uncertain how these two extracts cause genotoxicity. Nonetheless, it has been shown in several studies that saponins can lead to oxidative stress by increasing the levels of free radicals and reactive oxygen species (ROS). These radicals can disrupt DNA strands and cause mutations (Lv et al. 2013; Rahmouni et al. 2018; Saoudi et al. 2021). At higher doses, saponins play a critical role in cytotoxicity by disrupting cell membranes and inducing apoptosis through the formation of pores in the cell membrane (Podolak et al. 2010, 2023; Marrelli et al. 2016). While *Crocus sativus* corm extracts contain high levels of bioactive molecules, their potent antioxidant properties could contribute to cellular protection and disease prevention their potential genotoxic and cytotoxic effects at higher doses necessitate careful evaluation and dosage regulation to ensure safe usage.

This study provides valuable insights into the biochemical composition and biological effects of saffron corm extracts, there are still areas that warrant further investigation. Exploring the molecular mechanisms underlying the observed genotoxicity, along with additional in vivo studies, would help refine our understanding of how these bioactive compounds interact with biological systems. Furthermore, a more detailed assessment of how storage conditions influence the stability and bioavailability of these compounds could provide useful information for optimizing their potential applications.

## Conclusion

The results of these studies illustrate that HEES is rich in bioactive molecules and they have a higher antioxidant effect than HEEF, but both extracts are genotoxic and cytotoxic in the same higher doses. This effect necessitates a careful consideration of their use. Further investigation is required to clarify the mechanisms underlying these toxic effects and to optimize the safe and effective application of corm extracts in various fields. So, we can conclude that stocked *Crocus sativus* L. corms are a very interesting source of bioactive molecules that deserve to be valorized in the health field.

## Data Availability

All data supporting the findings of this study are available within the paper. Data generated are available from the corresponding authors upon request.
